# Resveratrol Inhibits Pancreatic Cancer Stem Cell Characteristics in Human and Kras^G12D^ Transgenic Mice by Inhibiting Pluripotency Maintaining Factors and Epithelial-Mesenchymal Transition

**DOI:** 10.1371/journal.pone.0016530

**Published:** 2011-01-31

**Authors:** Sharmila Shankar, Dara Nall, Su-Ni Tang, Daniel Meeker, Jenna Passarini, Jay Sharma, Rakesh K. Srivastava

**Affiliations:** 1 Department of Pathology and Laboratory Medicine, The University of Kansas Cancer Center, The University of Kansas Medical Center, Kansas City, Kansas, United States of America; 2 Department of Pharmacology, Toxicology and Therapeutics, and Medicine, The University of Kansas Cancer Center, The University of Kansas Medical Center, Kansas City, Kansas, United States of America; 3 Celprogen, San Pedro, California, United States of America; University of Pennsylvania, United States of America

## Abstract

**Background:**

Cancer stem cells (CSCs) can proliferate and self-renew extensively due to their ability to express anti-apoptotic and drug resistant proteins, thus sustaining tumor growth. Therefore, the strategy to eradicate CSCs might have significant clinical implications. The objectives of this study were to examine the molecular mechanisms by which resveratrol inhibits stem cell characteristics of pancreatic CSCs derived from human primary tumors and Kras^G12D^ transgenic mice.

**Methodology/Principal Findings:**

Human pancreatic CSCs (CD133^+^CD44^+^CD24^+^ESA^+^) are highly tumorigenic and form subcutaneous tumors in NOD/SCID mice. Human pancreatic CSCs expressing high levels of CD133, CD24, CD44, ESA, and aldehyde dehydrogenase also express significantly more Nanog, Oct-4, Notch1, MDR1 and ABCG2 than normal pancreatic tissues and primary pancreatic cancer cells. Similarly, CSCs from Kras^G12D^ mice express significantly higher levels of Nanog and Oct-4 than pancreatic tissues from Pdx-Cre mice. Resveratrol inhibits the growth (size and weight) and development (PanIN lesions) of pancreatic cancer in Kras^G12D^ mice. Resveratrol inhibits the self-renewal capacity of pancreatic CSCs derived from human primary tumors and Kras^G12D^ mice. Resveratrol induces apoptosis by activating capase-3/7 and inhibiting the expression of Bcl-2 and XIAP in human CSCs. Resveratrol inhibits pluripotency maintaining factors (Nanog, Sox-2, c-Myc and Oct-4) and drug resistance gene ABCG2 in CSCs. Inhibition of Nanog by shRNA enhances the inhibitory effects of resveratrol on self-renewal capacity of CSCs. Finally, resveratrol inhibits CSC's migration and invasion and markers of epithelial-mesenchymal transition (Zeb-1, Slug and Snail).

**Conclusions/Significance:**

These data suggest that resveratrol inhibits pancreatic cancer stem cell characteristics in human and Kras^G12D^ transgenic mice by inhibiting pluripotency maintaining factors and epithelial-mesenchymal transition. In conclusion, resveratrol can be used for the management of pancreatic cancer.

## Introduction

Pancreatic cancer is the fourth leading cause of cancer death in the United States. It is expected that approximately 32,000 Americans will die from pancreatic cancer this year. With an overall 5-year survival rate of 3% [1], pancreatic cancer has one of the poorest prognoses among all cancers [2]. Aside from its silent nature and tendency for late discovery, pancreatic cancer also shows unusual resistance to chemotherapy and radiation. Only 20% of pancreatic cancer patients are eligible for surgical resection [3]. The operations are very complex, and unless performed by surgeons specially trained and experienced in this procedure, they can be associated with very high rates of operative morbidity and mortality. Unfortunately, many pancreatic cancers are not resectable at the time of diagnosis. Furthermore, there are limited treatment options available for the patients with pancreatic cancer because chemo- and radio-therapies are largely ineffective, and metastatic disease frequently redevelops even after surgery. Therefore, there is an urgent need to discover novel and effective approaches for the prevention and/or treatment of pancreatic cancer.

It is now being realized that tumors contain a small number of tumor-forming and self-renewing cancer stem cells (CSCs) within a population of nontumor-forming cancer cells [4]. We and other have identified CSCs in several types of human cancers including pancreatic cancer [5], [6], [7], [8], [9]. Cancer stem cells hypothesis suggest that conventional chemotherapies kill differentiated or differentiating cells, and these cells form the bulk of the tumor, but can not generate new cells. Tumor relapse may occur because CSCs remain untouched, suggesting the removal of CSCs is very crucial for effective cancer therapy. Unlike most cells within the tumor, CSCs, including pancreatic CSCs, are resistant to chemotherapy and may contribute to tumor metastasis and tumor recurrence after treatment. Therefore, drugs that selectively target CSCs offer a greater promise for cancer therapy and/or prevention.

Epidemiological and dietary intervention studies in animals and humans have suggested that diet-derived phenolics, in particular the flavonoids, may play a beneficial role in inhibiting, reversing or retarding tumorigenesis in many types of cancers, including pancreatic cancer [10]. The polyphenolic compound resveratrol is a naturally occurring phytochemical and is found in many plant species, including grapes, peanuts and various herbs [10]. Resveratrol has been shown to have anti-inflammatory, antioxidant, antitumor, neuroprotective and immunomodulatory activities [10], [11], [12], [13], [14]. It also has activity in the regulation of multiple cellular events associated with carcinogenesis [10], [11], [12], [13], [14]. Its anticancer effects in pancreatic cancer include its ability to inhibit cell proliferation and angiogenesis, and induce apoptosis in pre-clinical studies [15], [16], [17], [18], [19], [20]. Resveratrol can enhance antitumor activity of gemcitabine *in vitro* and in orthotopic mouse model of human pancreatic cancer [19]. We and others have demonstrated that resveratrol enhances the therapeutic potential of anticancer drugs and sensitizes cancer cells to chemotherapy and radiotherapy [10], [19], [21], [22], [23]. Although these studies have examined the affect of resveratrol on pancreatic cancer, there are no studies examining how resveratrol regulates pancreatic cancer stem cell characteristics.

Epithelial cells undergo fibroblastoid morphological changes associated with increased motility or invasiveness due to decreased cell-cell adhesion (11–14). Fibroblastoid morphological changes of epithelial cells are known as epithelial-to-mesenchymal transition (EMT). EMT induction in cancer cells results in the acquisition of invasive and metastatic properties [24], [25], [26], [27], [28]. Recent reports indicate that the emergence of CSCs occurs in part as a result of EMT, for example, through cues from tumor stromal components. CSCs and EMT-type cells, which shares molecular characteristics with CSCs, have been believed to play critical roles in drug resistance and early cancer metastasis as demonstrated in several human malignancies including pancreatic cancer [27], [29], [30]. Thus, the discovery of molecular knowledge of drug resistance and metastasis in relation to CSCs and EMT in pancreatic cancer is becoming an important area of research, and such knowledge is likely to be helpful in the discovery of newer drugs as well as designing novel therapeutic strategies for the treatment and/or prevention of pancreatic cancer with better outcome.

The objectives of our study were to examine the molecular mechanisms by which resveratrol inhibits pancreatic CSC characteristics in human and Kras^G12D^ transgenic mouse model, and examine whether inhibition of Nanog enhances the ability of resveratrol to inhibit self-renewal capacity of human pancreatic CSCs.

## Results

### Isolation and characterization of human pancreatic CSCs isolated from human primary pancreatic tumors

**Figure 1:**
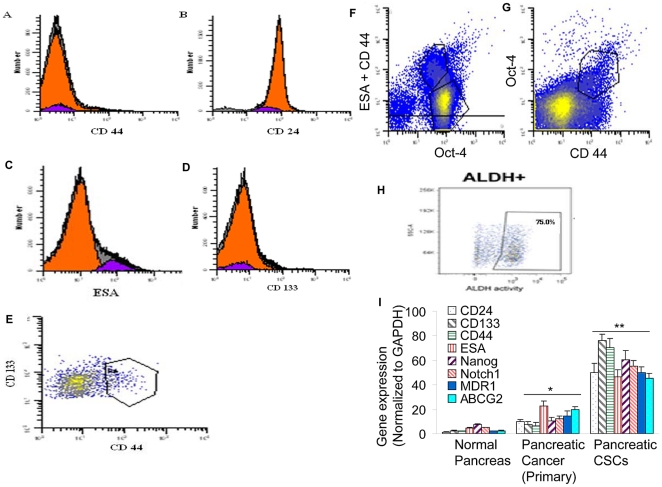
Isolation and characterization of human pancreatic CSCs from human primary tumors. (A–H), Pancreatic cancer cells were isolated from primary tumors and analyzed by flow cytometry using antibody against CD44, CD24, ESA, CD133, Oct4, and ALDH. (I), Expression of stem cell markers. RNA was isolated from normal pancreatic tissues, primary pancreatic cancer and pancreatic CSCs and the expression of CD24, CD133, CD44, ESA, Nanog, Notch1, MDR1 and ABCG2 was measured by q-RT-PCR. Data represent mean ± SD. *, ** @  =  significantly different from controls, P<0.05.

Tumors contain a small number of tumor-forming and self-renewing CSCs within a population of nontumor-forming cancer cells [4]. Therapeutic failure/recurrence is due to ineffective targeting of CSC population. The CD44^+^CD24^+^ESA^+^ pancreatic cancer cells are highly tumorigenic and possess the stem cell-like properties of self-renewal and the ability to produce differentiated progeny [31]. From a therapeutic point of view, CSCs in several tumor types have shown resistance to standard therapies and may play a role in treatment failure or disease recurrence. Identification of pancreatic CSCs and further elucidation of the signaling pathways that regulate their growth and survival may provide novel therapeutic and preventive approaches for the management of pancreatic cancer. We first characterized CSCs isolated from human pancreatic tumors (Fig. 1A–H). Pancreatic CSCs express stem cell markers CD44, ESA, CD133, and CD24, and pluripotency maintaining factor Oct-4. Pancreatic CD44^+^ESA^+^CD133^+^CD24^+^ CSCs also expressed ALDH (aldehyde dehydrogenase), another marker for CSCs.

We next examined the expression of stem cell markers (CD133^+^CD44^+^CD24^+^ESA^+^), pluripotency maintaining factors (Nanog), signaling molecule (Notch-1) and drug resistant genes (MDR1 and ABCG2) in human normal pancreas, primary pancreatic cancer cells, and CSCs by q-RT-PCR (Fig. 1I). Normal pancreatic tissues express very low levels of CD133, CD24, CD44, ESA, Nanog, Notch1, MDR1 and ABCG2 compared to pancreatic cancer and CSCs. Human pancreatic tumors expressed about 3–6% CSCs. By comparison, the expression of these genes in pancreatic cancer cells was significantly lower than CSCs. Interestingly, CD133^+^CD44^+^CD24^+^ESA^+^ CSC express higher levels of Nanog, Notch1, MDR1 and ABCG2 genes.

### Tumorigenic potential of CD133^+^CD44^+^CD24^-^ESA^+^ pancreatic CSCs in NOD/SCID mice

**Figure 2:**
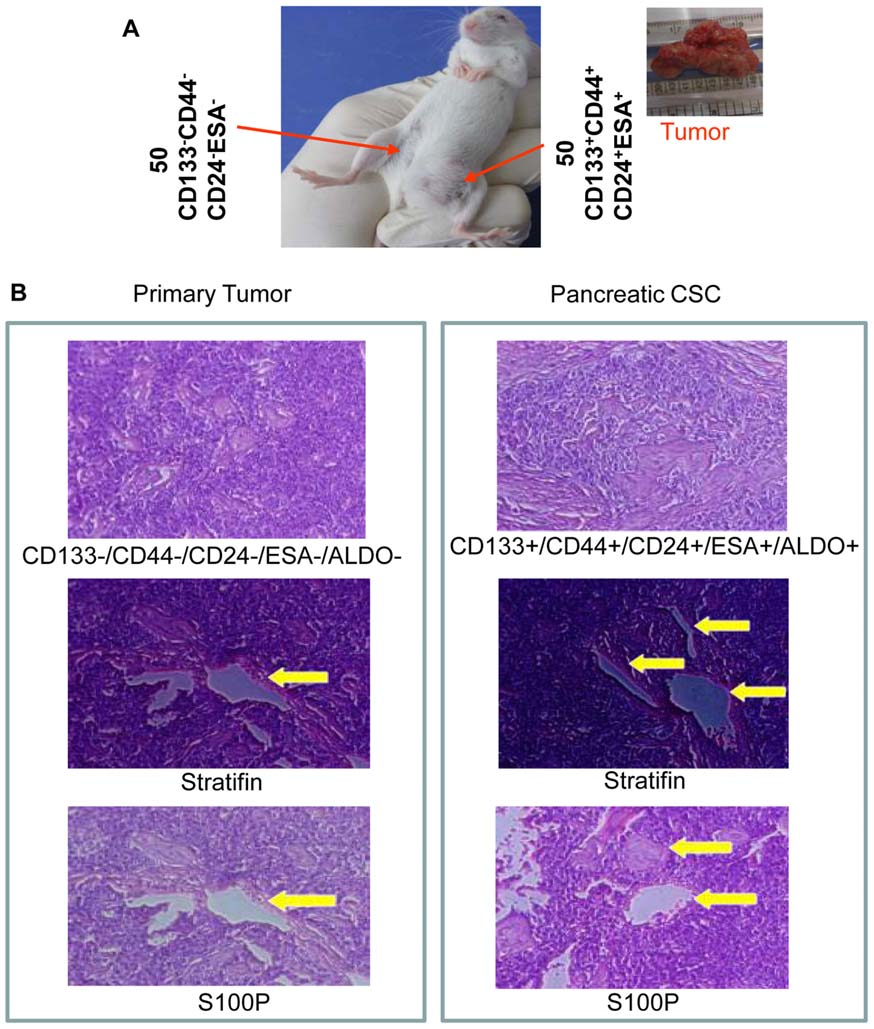
Tumorigenic potential of pancreatic CSCs in NOD/SCID mice. (A), NOD/SCID mice were sc injected with 50 CD133^−^CD44^−^CD24^−^ESA^−^ cells on right flank and CD133^+^CD44^+^CD24^+^ESA^+^ CSCs on left flanks and tumor growth was observed for 20 days. (B), Pancreatic CSCs are similar to human primary tumors. Tumor sections from human primary pancreatic tumors, and pancreatic CSCs grown in NOD/SCID mice were stained with H&E, Statifin and S100P.

We next compared the tumorigenic potential of CD133^−^CD44^−^CD24^−^ESA^−^ pancreatic CSCs with that of CD133^+^CD44^+^CD24^−^ESA^+^ pancreatic CSCs isolated from human primary tumors in NOD/SCID mice (Fig. 2). Nude mice were injected with 50 CD44^−^CD44^−^CD24^−^ESA^−^ CSCs and CD133^+^CD44^+^CD24^+^ESA^+^ CSCs in left and right flanks, respectively. The data demonstrated that CD133^+^CD44^+^CD24^+^ESA^+^ CSCs formed tumors in 20 days in NOD/SCID mice, whereas CD133^−^CD44^−^CD24^−^ESA^−^ CSCs did not form tumors (Fig. 2A). H&E staining of CD44^+^CD24^+^ESA^+^ CSC tumors formed in nude mice revealed similar tumor phenotype with that of primary human tumors (Fig. 2B). The expressions of S100P and Stratifin were also similar in primary tumors and pancreatic CSCs. These data suggest that CD133^+^CD44^+^CD24^+^ESA^+^ CSCs are tumorigenic, and posses CSC characteristics.

### Resveratrol inhibits the formation of primary and secondary spheroids and cell viability of human pancreatic CSCs

**Figure 3:**
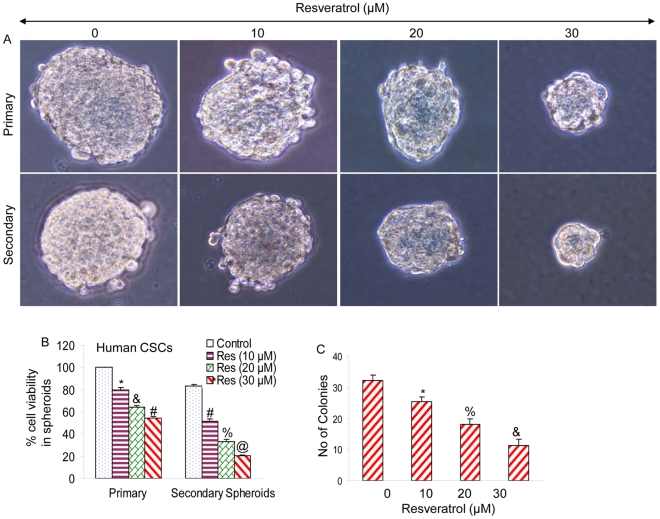
Resveratrol inhibits cell viability in spheroids and colony formation by pancreatic CSCs. (A), Human pancreatic CSCs were grown in six-well ultralow attachment plates (Corning Inc., Corning, NY) at a density of 1,000 cells/ml in Celprogen pancreatic CSC medium at 37°C in a humidified atmosphere of 95% air and 5% CO_2_ and treated with resveratrol (0–30 µM) for 7 days to obtain primary spheroids. At the end of incubation period, spheroids were collected, reseeded and treated with resveratrol for another week to obtain secondary spheroids. (B) Cell viability in spheroids was measured by trypan blue assay at the end of 7 and 14 days from above experiment. Data represent mean ± SD. *, &, #, % and @  =  significantly different from controls, P<0.05. (C), Pancreatic CSCs were seeded in soft agar and treated with resveratrol (0–30 µM) for 21 days. At the end of incubation period, numbers of colonies were counted. Data represent mean ± SD. *, % and &  =  significantly different from control, P<0.05.

CSCs were grown in pancreatic cancer stem cell defined medium in suspension, and treated with resveratrol for 7 days. At the end of incubation period, spheroids in each well were photographed. Resveratrol inhibited the growth of spheroids in suspension in a dose-dependent manner (Fig. 3A). The spheroids from each treatment group were collected and resuspended for counting cell viability. Resveratrol inhibited pancreatic CSC viability in a dose-dependent manner (Fig. 3B).

Since resveratrol inhibited the growth of tumor spheroid and cell viability of CSCs, we next sought to examine the effects of resveratrol on colony formation. Pancreatic CSCs were grown in agar, and treated with various doses of resveratrol for 3 weeks. At the end of incubation period, colonies were counted. Resveratrol inhibited the growth of colonies in a dose-dependent manner (Fig. 3C). These data suggest that resveratrol can be effective in inhibiting the self-renewal capacity of human pancreatic CSCs.

### Resveratrol activates caspase-3/-7, induces apoptosis, and inhibits the expression of XIAP, Bcl-2 and cyclin D1 in pancreatic CSCs

**Figure 4:**
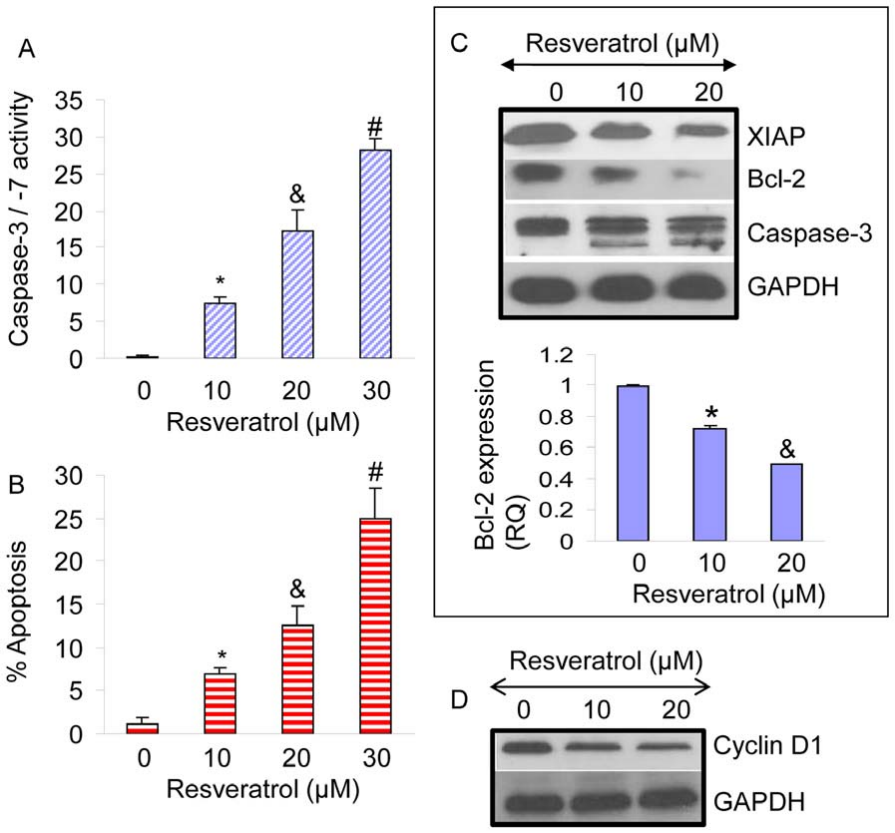
Resveratrol activates caspase-3/-7, induces apoptosis, and inhibits the expression of XIAP, Bcl-2 and cyclin D1 in pancreatic CSCs. (A and B), Activation of caspase-3/-7 and induction of apoptosis. Pancreatic CSCs were treated with resveratrol (0–30 µM) for 48 h, and caspase-3/-7 activity and apoptosis were measured by colorometric and TUNEL assay, respectively. Data represent mean ± SD. *, & and #  =  significantly different from control, P<0.05. (C and D), Pancreatic CSCs were treated with resveratrol (0–20 µM) for 48 h, and western blot analysis was performed to measure the expression of XIAP, Bcl-2, caspase-3 and cyclin D1. GAPDH was used as a loading control.

We next examined the effects of resveratrol on caspase-3/-7 activity, apoptosis, and expression of apoptosis- and cell cycle-related proteins (Fig. 4). Resveratrol induces caspase-3/-7 activity and apoptosis (Fig. 4A and B). Since IAPs, Bcl-2 family members and cyclin D1 play major roles in regulation of apoptosis and cell cycle, we examined the effects of resveratrol on the expression of XIAP, Bcl-2, caspase-3 and cyclin D1. Resveratrol inhibited the expression of XIAP, Bcl-2 and cyclin D1, and also cleaved caspase-3 (Fig. 4C and D). These data suggest that resveratrol causes growth arrest and induces apoptosis in pancreatic CSCs through inhibition of cyclin D1 and apoptosis-related proteins (XIAP and Bcl-2), respectively.

### Resveratrol inhibits the expression of Nanog, Sox-2, cMyc, Oct-4 and ABCG2 in human pancreatic CSCs

**Figure 5:**
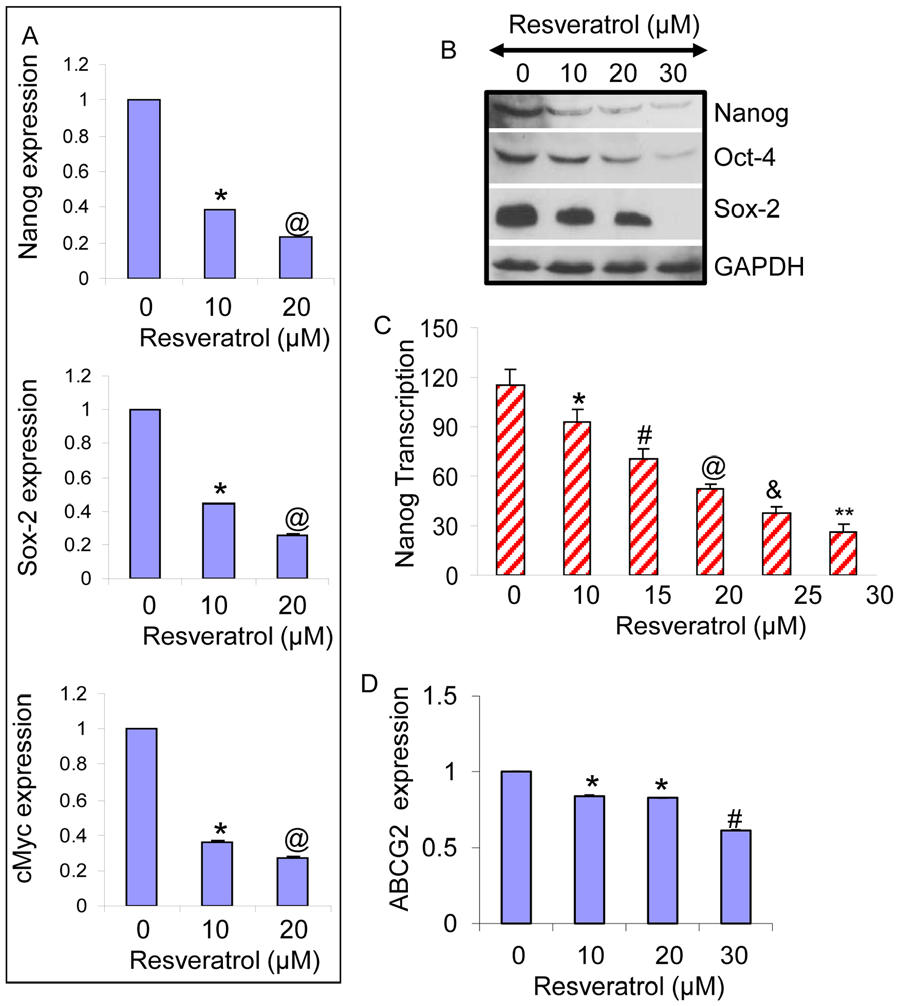
Inhibition of Nanog, Sox-2, cMyc, Oct-4 and ABCG2 in human pancreatic CSCs by resveratrol. (A), Pancreatic CSCs were treated with resveratrol (0–20 µM) for 24 h. The expression of Nanog, Sox-2 and cMyc was measured by qRT-PCR. Data represent mean ± SD. * and @  =  significantly different from control, P<0.05. (B), Pancreatic CSCs were treated with resveratrol (0–30 µM) for 48 h. The expression of Nanog, Oct-4 and Sox-2 was measured by the Western blot analysis. GAPDH was used as a loading control. (C), Inhibition of Nanog transcription by resveratrol. Pancreatic CSCs were transduced with Nanog reporter construct. Transduced cells were treated with resveratrol to examine the Nanog transcriptional activity. Data represent mean ± SD. *, #, @, & and **  =  significantly different from control, P<0.05. (D), Pancreatic CSCs were treated with resveratrol (0–30 µM) for 36 h. The expression of ABCG2 was measured by qRT-PCR. Data represent mean ± SD. * and #  =  significantly different from control, P<0.05.

Since transcription factors Nanog, Sox-2, cMyc and Oct-4 are highly expressed in CSCs and are required for maintaining pluripotency, we examined the effects of resveratrol on the expression of Nanog, Sox-2, cMyc and Oct-4 in human pancreatic CSCs expressing CD44^+^/CD24^+^/ESA^+^ (Fig. 5). Resveratrol inhibited the expression of Nanog, Sox-2 and cMyc as measured by qRT-PCR (Fig. 5A). Similarly, resveratrol inhibited the expression of Nanog, Oct-4 and Sox-2 in pancreatic CSCs as measured by the Western blotting (Fig. 5B). Resveratrol also inhibited the Nanog reporter activity (Fig. 5C). These data suggest that resveratrol inhibits the factors required for maintaining pluripotency in pancreatic CSCs.

Cancer stem cells have been shown to exhibit drug resistance properties by expressing multidrug resistance genes such as ABCG2. We therefore measured the expression of ABCG2 in human CSCs treated with resveratrol. Resveratrol inhibited the expression of ABCG2 in pancreatic CSCs as measured by qRT-PCR (Fig. 5D). These data suggest that inhibition of ABCG2 by resveratrol could be beneficial in enhancing the biological effects of resveratrol alone or in combination with other drugs.

### Nanog shRNA enhances the inhibitory effects of resveratrol on pancreatic CSC viability in spheroids

**Figure 6:**
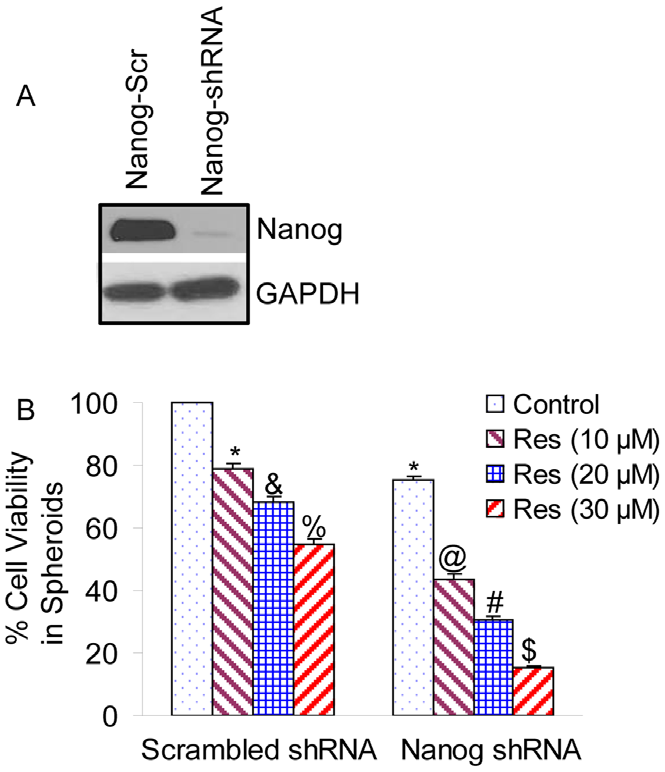
Nanog shRNA enhances the inhibitory effects of resveratrol on self-renewal capacity of pancreatic cancer stem cells. (A), Expression of Nanog. Pancreatic CSCs were transduced with scrambled or Nanog shRNA, and Western blot analyses were performed to examine the expression of Nanog and GAPDH. (B), Transduced cells were treated with resveratrol (0–30 µM) and spheres in suspension were grown for one week. Spheres were harvested, resuspended and cell viability was determined by trypan blue assay.

Since Nanog is highly expressed in pancreatic CSCs, we sought to examine whether inhibition of Nanog enhances the biological effects of resveratrol on stem cell viability in spheroids. Transduction of Nanog shRNA in CSCs inhibited Nanog expression, as shown by the western blot analysis (Fig. 6A). Resveratrol inhibited cell viability in spheroids (scrambled shRNA) in a dose-dependent manner (Fig. 6B). Transduction of Nanog shRNA further enhanced the inhibitory effects of resveratrol on cell viability in spheroids. These data suggest that inhibition of Nanog can enhance the chemopreventive effects of resveratrol.

### Resveratrol inhibits markers of epithelial-mesenchymal transition, invasion and migration in human pancreatic CSCs

**Figure 7:**
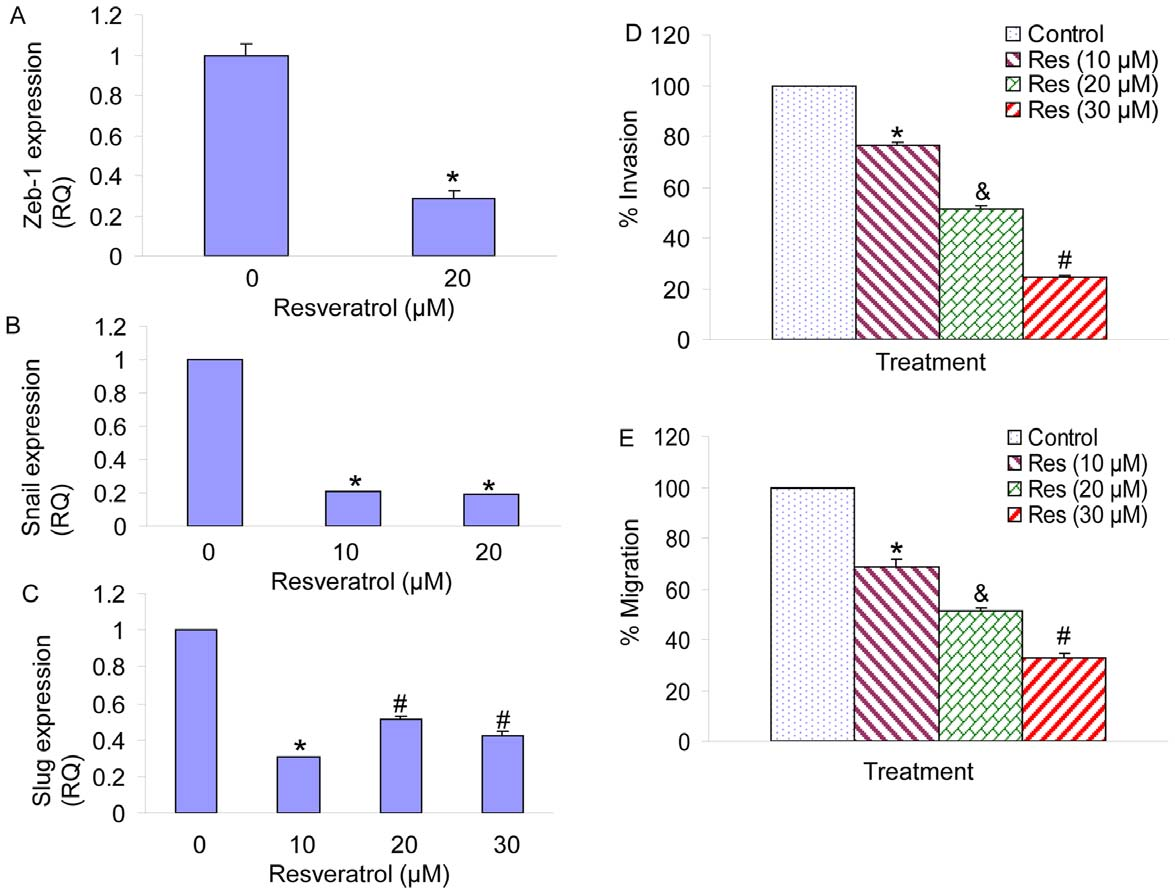
Regulation of EMT markers, invasion and migration by resveratrol in pancreatic CSCs. Pancreatic CSCs were treated with resveratrol (0–0 µM) for 24 h. The expression of Zeb-1 (A), Snail (B) and Slug (c) was measured by the qRT-PCR. Data represent the mean ± S.D. * or #  =  significantly different from respective controls, P<0.05. (D), Invasion assay. Pancreatic CSCs were plated onto the Matrigel-coated membrane in the top chamber of the transwell and treated with resveratrol (0–30 µM) for 48 h. Cells invaded to the lower chambered were fixed with methanol, stained with crystal violet and counted. Data represent mean ± SD. *, & or #  =  significantly different from control, P<0.05. (E) Migration assay. Pancreatic CSCs were plated in the top chamber of the transwell and treated with resveratrol (0–30 µM) for 24 h. Cells migrated to the lower chambered were fixed with methanol, stained with crystal violet and counted. Data represent mean ± SD. *, & or #  =  significantly different from control, P<0.05.

Epithelial to mesenchymal transitions (EMT) are transdifferentiation programs that are required for tissue morphogenesis during embryonic development [32]. The EMT process can be regulated by a diverse array of cytokines and growth factors, such as TGFβ, whose activities are dysregulated during malignant tumor progression [33]. Thus, EMT induction in cancer cells results in the acquisition of invasive and metastatic properties. Recent reports indicate that the emergence of CSCs occurs in part as a result of EMT, for example, through cues from tumor stromal components. EMT plays a crucial role in tumorigenesis, cancer progression and drug resistance [28], [32], [34]. Recent studies revealed that there is a direct link between the EMT program and the gain of epithelial stem cell properties [28], [32], [34], [35]. EMT is sufficient to induce a population with stem cell characteristics from well-differentiated epithelial cells and cancer cells. We next examined the effects of resveratrol on the expression of EMT markers in pancreatic CSCs (Fig. 7). Resveratrol inhibited the expression of Zeb-1, Snail and Slug. Resveratrol also inhibited invasion and migration of pancreatic CSCs (Fig. 7D and E). These data suggest that resveratrol can regulate EMT, invasion and migration by inhibiting the expression of Zeb-1, Snail and Slug in CSCs.

### Resveratrol inhibits the growth and development of pancreatic cancer in Kras^G12D^ mice

**Figure 8:**
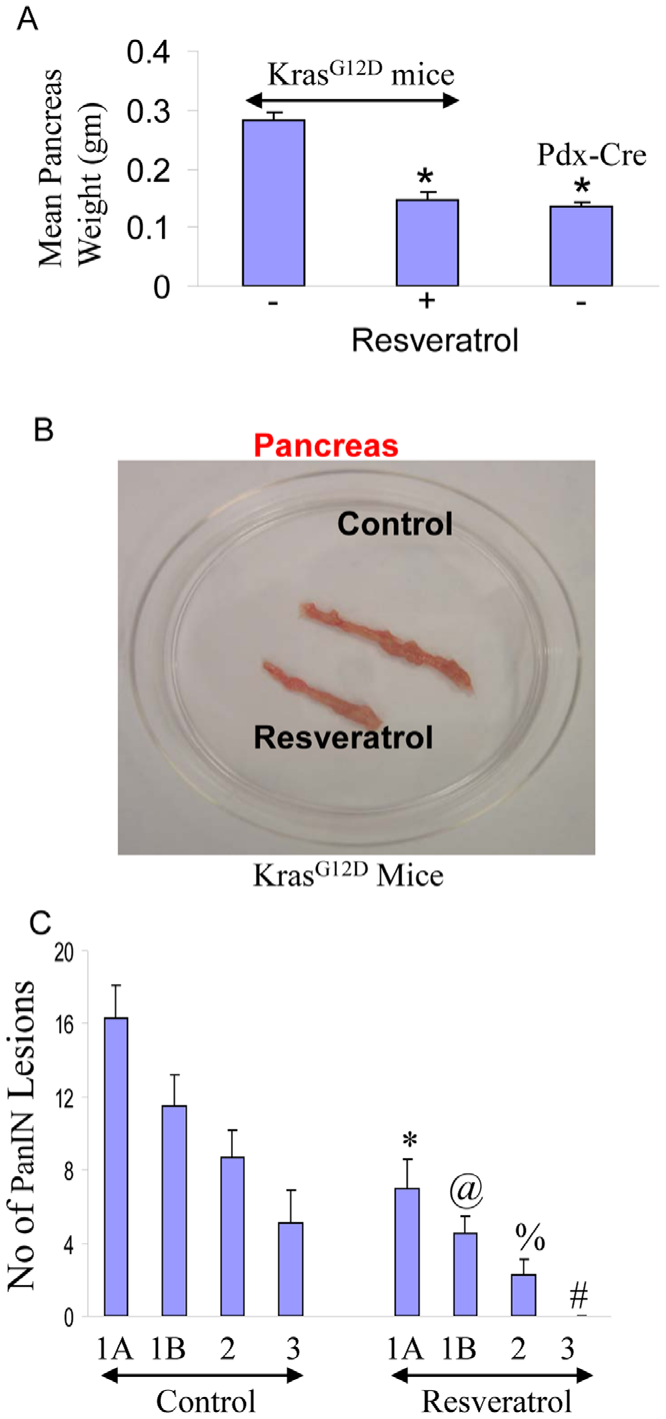
Resveratrol inhibits pancreatic cancer growth and development in Kras^G12D^ mice, and pancreatic CSC characteristics. (A), Inhibition of pancreatic cancer growth by resveratrol. Kras^G12D^ transgenic mice were treated with resveratrol (40 mg/kg) by gavage once everyday (Monday – Friday) for about 10 months (mice age 12 months). Pancreas weights from control and resveratrol treated mice were taken. Weight of pancreas from Pdx-cre mice was also taken. (B) Picture of pancreas obtained from control and resveratrol treated Kras^G12D^ mice. (C), Quantification of PanIN lesions in the pancreas of control and resveratrol treated Kras^G12D^ mice. Data represent the mean ± S.D. *, @, %, #  =  significantly different from respective controls, P<0.05.

Pancreatic ductal adenocarcinoma (PDAC) is characterized by mutations in Kras and frequent deregulation of crucial embryonic signaling pathways. The Kras*^G12D^* mice develop spontaneous tumors in the pancreas, and it truly resembles the growth of human pancreatic cancers [36]. In *Pdx1-Cre;LSL-Kras^G12D^* (*Kras^G12D^*) mice, physiological levels of *Kras^G12D^* induce ductal lesions that recapitulate the full spectrum of human PanINs, putative precursors to invasive pancreatic cancer. We therefore examined the effects of resveratrol on pancreatic carcinogenesis in Kras^G12D^ mice (Fig. 8). Resveratrol inhibited the growth of pancreas (weight and size) in Kras^G12D^ mice compared to that of untreated control (Fig. 8A and B). The mean weight of pancreas from Kras^G12D^ mice was similar to that of Pdx1-Cre mice.

Quantifications of PanIN lesions in pancreas from Kras^G12D^ mice treated with or without resveratrol for about 10 months (age about 12 months) are shown in Fig. 8C. Untreated (control) Kras^G12D^ mice showed various stages of cancer development ranging from PanIN-1A to PanIN-3 stages. By comparison, treatment of Kras^G12D^ mice with resveratrol significantly inhibited pancreatic cancer development as evident by reduced numbers of PanIN-1A, PanIN-1B and PanIN-2 lesions. Interestingly, resveratrol treated Kras^G12D^ mice showed no PanIN 3 lesions. These data suggest that resveratrol inhibits the growth and development of pancreatic cancer in Kras^G12D^ transgenic mice.

### Resveratrol inhibits self-renewal capacity of pancreatic CSCs

**Figure 9:**
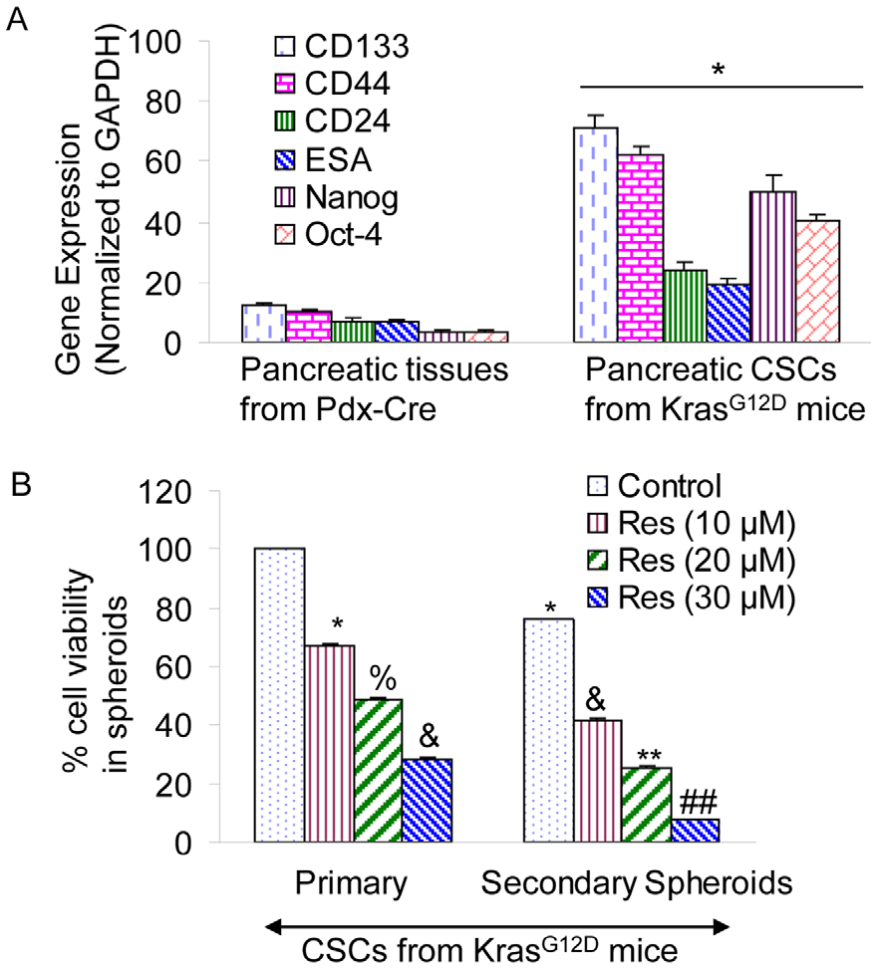
Resveratrol inhibits the pancreatic cancer stem cell characteristics Kras^G12D^ mice. (A), Pancreatic CSCs were isolated from Kras^G12D^ mice (about 12 months old) using CD133, CD44, CD24, and ESA antibodies. The expression of these markers and Nanog and Oct-4 was confirmed by qRT-PCR. Data represent mean ± SD. *  =  significantly different from the same gene of Pdx-Cre mice at P<0.05. (B), CSCs were grown in suspension and treated with resveratrol (0–30 µM) for 7 days to obtain primary spheroids. At the end of incubation period, spheroids were collected, resuspended and treated with resveratrol for another week to obtain secondary spheroids. Cell viability in spheroids was measured by trypan blue assay at the end of 7 and 14 days. Data represent mean ± SD. *, %, &, ** and ##  =  significantly different from respective controls, P<0.05.

We next examined whether the pancreas of Kras^G12D^ transgenic mouse harbor cancer stem cells, and whether resveratrol inhibits pancreatic CSCs in these mice. Treatment of Kras^G12D^ mice with resveratrol completely eradicated pancreatic CSCs in Kras^G12D^ mice at 12 months of age. Pancreatic CSCs isolated from Kras^G12D^ mice express high levels of stem cell markers (CD133, CD44, CD24 and ESA) and transcription factors (Nanog and Oct-4) (Fig. 9A). The expression of these markers in untreated Kras^G12D^ was significantly higher than Pdx-Cre mice. Pancreatic tumors expressed about 1.3–2.5% CSCs isolated from 12 months old Kras^G12D^ (control/untreated) mice. Since we have not seen any pancreatic CSCs in resveratrol treated mice, we sought to examine whether CSCs from control/untreated Kras^G12D^ mice are capable of self-renewing and respond to resveratrol *in vitro* (Fig. 9B). Resveratrol inhibited the self-renewal capacity of pancreatic CSCs as measured by formation of primary and secondary spheroids in suspension, and cell viability in those spheroids. These data suggest that resveratrol is effective in inhibiting the self-renewal capacity of pancreatic CSCs derived from Kras^G12D^ mice.

## Discussion

Cancer stem cells (CSCs) have been identified in a growing number of malignancies and are functionally defined by their ability to undergo self-renewal and produce differentiated progeny [37], [38]. These properties allow CSCs to recapitulate the original tumor when injected into immunocompromised mice. CSCs within an epithelial malignancy were first described in breast cancer and found to display specific cell surface antigen expression (CD44+CD24low/-) [39]. Since then, CSCs have been identified in an increasing number of other human malignancies using CD44 and CD24 as well as a number of other surface antigens. Physiologic properties, including aldehyde dehydrogenase (ALDH) activity, have also been used to isolate CSCs from malignant tissues [31], [39], [40], [41], [42], [43], [44]. Stem cells gain the quiescent phenotype by regulating cell cycle proteins and pluripotent stemness factors. Cancer stem/progenitor cells may exhibit characteristics similar to normal stem cells. Thus, stem cells are attractive candidates for cancer-initiating cells since their longevity and self-renewal capability may result in the accumulation of genetic mutations.

Our study demonstrates, for the first time, that cancer preventive agent resveratrol can inhibit the self-renewal capacity of pancreatic cancer stem cells derived from human primary tumors and Kras^G12D^ mice in vitro. We were unable to observe any pancreatic cancer stem cells in Kras^G12D^ mice treated with resveratrol. However, resveratrol inhibited the formation of primary and secondary spheroids in pancreatic cancer stem cells isolated from Kras^G12D^ mice *in vitro*. Resveratrol induces apoptosis by activating capase-3/7 and inhibiting the expression of Bcl-2, and XIAP in CSCs. Interestingly, resveratrol also inhibits the expression of ABCG2, a multidrug resistance gene which has been demonstrated to overexpress in CSCs. Furthermore, resveratrol inhibits epithelial-mesenchymal transition by inhibiting the expression of vimentin, slug and snail, and also retards CSC's migration and invasion. These data suggest that resveratrol can be used to prevent pancreatic cancer.

We have recently demonstrated that sulforaphane inhibited self-renewal capacity of pancreatic cancer stem cells as measured by spheroid and colony formation [7]. Sulforaphane inhibited the expression of pluripotent transcription factors (Nanog, Oct-4 and Sox-2). Inhibition of Nanog by lentiviral-mediated shRNA expression enhanced the self-renewal capacity of sulforaphane. Sulforaphane induced apoptosis by inhibiting the expression of Bcl-2 and XIAP, phosphorylation of FKFR, and activating caspase-3 [7]. Moreover, sulforaphane inhibited expression of proteins involved in the epithelial-mesenchymal transition (β-catenin, vimentin, twist-1, and ZEB1), suggesting the blockade of signaling involved in early metastasis. Furthermore, combination of quercetin with sulforaphane, had synergistic effects on self-renewal capacity of pancreatic CSCs. These data suggest that sulforaphane either alone or in combination with quercetin can eliminate cancer stem cell-characteristics. Similarly in the present study, resveratrol inhibited self-renewal capacity of pancreatic CSCs.

CSCs share principle characteristics with adult stem cells namely self-renewal, high proliferative potential, clonogenicity, and multipotency [45], [46], [47]. In addition, they have the ability to reproducibly form the same tumor phenotype as in the patient and to undergo differentiation into non-tumorigenic cells [48], [49], [50]. CSCs were first isolated from patients with hematologic malignancies in which a few cells could initiate a new tumor [51]. During the past few years, CSCs were also identified and isolated from solid tumors such as breast, brain, colon, pancreatic, and prostate tumors [29], [39], [44], [52], [53], [54], [55], [56]. In pancreatic cancer, CD44+/CD133+ pancreatic CSCs been identified [57], [58]. Because of the observed heterogeneity in pancreatic cancer, the use of single-cell markers for the selection, characterization/identification, and functional evaluation of stem/progenitor-like pancreatic cancer cells will not be appropriate. Deregulation of ALDH enzyme activity is implicated in the pathophysiology of various hematologic and epithelial cancers [40], [59], [60], [61], [62], [63], [64]. The introduction of FACS-based viable cell sorting for ALDH activity (ALDEFLUOR assays) in tumor biology has further substantiated a role of ALDH^hi^ subpopulations of cancer cells in carcinogenesis [40], [54], [60], [65]. High ALDH activity, as detected by the ALDEFLUOR assay, can thus be used as a functional marker to isolate CSCs in several types of epithelial cancers, including those of ovarian, breast, lung, pancreatic and colon [40], [54], [60], [63], [65], [66]. Recently, CSCs from pancreatic adenocarcinoma based on ALDH activity and the expression of the CD44 and CD24, and CD133 have been identified [31], [43], [67], [68]. These highly tumorigenic populations may or may not be overlapping and display other functions. It has been reported that ALDH(+) and CD44(+)CD24(+) pancreatic CSCs are similarly tumorigenic, but ALDH(+) cells are relatively more invasive and possess metastatic behavior [68]. Similarly in the present study, we have been able to isolate CD133, CD44, CD24, ESA and ALDH positive pancreatic CSCs and demonstrated their tumorigenic potential in NOD/SCID mice. The cancer stem cell hypothesis has provided a paradigm shift in our understanding of carcinogenesis, metastasis, and tumor biology. The identification of CSCs has important implications for designing new therapeutic approaches for the treatment and prevention of cancer. The involvement of CSCs in the formation of distant metastases, tumor dormancy and therapy resistance offers high hopes for treating cancer patients.

Recent evidence suggests a shared genomic fingerprint between embryonic stem cells, cancer cells, and cancer stem cells. Activation targets of Nanog, Oct-4, Sox2 and c-Myc are more frequently overexpressed in certain tumors. In the absence of bona fide cancer stem cell lines, human embryonic stem cells, which have similar properties to cancer and cancer stem cells, have been an excellent model throwing light on the anticancer affects of various putative anticancer agents. Nanog, Sox2 and Oct-4 are transcription factors which are essential to maintaining the pluripotent embryonic stem cell phenotype. Oct-4 and Sox2 bind to the Nanog promoter in living mouse and human ESCs [69]. Nanog, Oct-4 and Sox2 co-occupy and regulate their own promoters together with other developmental genes with diverse functions and collaborate to form an extensive regulatory circuitry including autoregulatory and feed-forward loops [69], [70], [71]. A high level of Nanog is a key regulator of ESC self-renewal and puripotency. Nanog-deficient ES cells and embryos lose their pluripotency [72]. Nanog overexpression leads to the clonal expansion of ES cells through circumvention of the LIF-dependent Stat-3 pathway and sustained Oct-4 expression levels [72], [73]. Genome-wide gene expression profiling shows that Nanog is expressed at high levels in testicular carcinoma in situ and germ cell tumors [74]. Positive correlations of Oct-4, Nanog, or CD133 expression on tumor stage were shown on oral squamous cell carcinoma patient tissues [75]. We have recently demonstrated that inhibition of Nanog by shRNA enhanced the inhibitory effects of EGCG and SFN on self-renewal capacity of CSCs, suggesting its requirement for self-renewal of CSCs [6], [7]. Medulloblastoma-associated CSCs selected by serum-free medium with bFGF and EGF can form 3D spheroids and display enhanced self-renewal and highly co-expressed stem cell genes (Oct4, Nanog, Nestin, and Musashi-1) as well as anti-apoptotic genes (Bcl-2 and Bcl-X_L_) [76]. These finding suggest that inhibition of pluripotency maintaining transcription factor such as Nanog could be a novel strategy to kill CSCs.

During EMT, epithelial cells lose their characteristics and gain mesenchymal features. It has been suggested that transformed epithelial cells can activate embryonic programs of epithelial plasticity and switch from a sessile, epithelial phenotype to a motile, mesenchymal phenotype. Induction of EMT can, therefore, lead to invasion of surrounding stroma, intravasation, dissemination and colonization of distant sites. Under the cancer stem cell hypothesis, sustained metastatic growth requires the dissemination of a CSC from the primary tumor followed by its re-establishment in a secondary site. SNAI, ZEB and TWIST family members repress the *CDH1* gene to induce EMT, but also regulate the transcription of other target genes. TWIST1 is upregulated in human breast cancer, gastric cancer, esophageal cancer, and prostate cancer. E-cadherin, occludin and cytokeratin are downregulated during EMT, while N-cadherin, vimentin, fibronectin, SNAI1/SAIL, SNAI2/SLUG, ZEB2/SIP1, and TWIST1 are upregulated [77]. E-cadherin and N-cadherin are representative adhesion molecules expressed on epithelial cells and mesenchymal cells, respectively. E-cadherin at the adherens junction is implicated in the stable cell-cell contact of epithelial cells, while N-cadherin in the weak intercellular contact of mesenchymal cells [78], [79]. Class switch from E-cadherin to N-cadherin results in the loss of epithelial phenotype and the acquisition of mesenchymal phenotype. Transcriptional repression of *CDH1* gene or functional repression of E-cadherin protein is the critical step for EMT. Upregulation of EMT regulators is associated with more malignant phenotypes in a variety of human cancer, such as gastric cancer, pancreatic cancer, breast cancer, and ovarian cancer. In the present study, resveratrol inhibited the expression of Snail, Slug and Zeb-1 in pancreatic cancer stem cells. Similarly in our other studies, we have demonstrated that EGCG and sulforaphane inhibited the expression of EMT markers (expression of vimentin, nuclear β-catenin and TCF-1/LEF reporter activity) and also inhibited the transcription factors slug and snail [6], [7]. The inhibition of EMT markers by these agents suggests that they could inhibit early metastasis of pancreatic CSCs.

Pancreatic ductal adenocarcinoma (PDAC) is characterized by near-universal mutations in Kras and frequent deregulation of crucial embryonic signaling pathways. The *Kras^G12D^* mice develop spontaneous tumors in the pancreas, and it truly resembles the growth of human pancreatic cancers [36]. In *Pdx1-Cre;LSL-Kras^G12D^* (*Kras^G12D^*) mice, physiological levels of *Kras^G12D^* induce ductal lesions that recapitulate the full spectrum of human PanINs, putative precursors to invasive pancreatic cancer. In the present study using KrasG12D mice, resveratrol inhibited the weight of pancreas, which was similar to that of Pdx1-Cre mice. Furthermore, untreated (control) Kras^G12D^ transgenic mice at 12 months of age showed various stages of cancer development ranging from PanIN-1A to PanIN-3 stages. By comparison, treatment of Kras^G12D^ mice with resveratrol significantly inhibited pancreatic cancer development as evident by reduced numbers of PanIN-1A, PanIN-1B and PanIN-2 lesions. Interestingly, resveratrol treated Kras^G12D^ mice showed no PanIN 3 lesions. Pancreatic CSCs isolated from Kras^G12D^ mice express high levels of stem cell markers (CD133, CD44, CD24 and ESA) and transcription factors (Nanog and Oct-4). The expression of these markers in untreated Kras^G12D^ was significantly higher than Pdx-Cre mice. We were unable to detect any CSCs in resveratrol treated Kras^G12D^ Mice. However, resveratrol inhibited the self-renewal capacity of pancreatic CSCs isolated from Kras^G12D^ mice, as measured by formation of primary and secondary spheroids in suspension, and cell viability in those spheroids. These data suggest that resveratrol (i) inhibits growth (weight and size) and development (PanINS stages) of pancreatic cancer in Kras^G12D^ transgenic mice, and (ii) inhibits the self-renewal capacity of pancreatic CSCs derived from Kras^G12D^ mice.

In conclusion, we have demonstrated that resveratrol inhibited self-renewal capacity of pancreatic CSCs isolated from human primary cancer and Kras^G12D^ mice, Resveratrol inhibited the pancreatic cancer growth and development (PanIN lesions) in Kras^G12D^ mice, and also inhibited the expression of transcription factors which are required for maintaining pluripotency. Furthermore, inhibition of Nanog could be considered as a novel strategy to enhance the biological effects of anticancer and chemopreventive agents or sensitize those cells which are resistant to chemotherapy or irradiation. Moreover, resveratrol inhibited invasion, migration and the expression of proteins involved in the EMT, suggesting the blockade of signaling involved in early metastasis. These data suggest that resveratrol can be used for the prevention and/or treatment of pancreatic cancer.

## Methods

### Ethics statement

Experiments were conducted under approved protocols by the Institutional Animal Care and Use Committee (IACUC) at the University of Texas Health Science Center at Tyler (protocol #372), and at Celprogen (protocol IRB # 1070080-00).

### Reagents

Antibodies against vimentin, slug, snail, GAPDH, XIAP, caspase-3, cyclin D1, Bcl-2, Oct-4, Sox-2, and Nanog were purchased from Cell Signaling Technology, Inc. (Danvers, MA). Resveratrol was purchased from LKT Laboratories, Inc. (St. Paul, MN). The Aldefluor assay kit was purchased from Stem Cell Technologies (Vancouver, BC). Enhanced chemiluminescence (ECL) Western blot detection reagents were purchased from Amersham Life Sciences Inc. (Arlington Heights, IL). All other chemicals were purchased from Sigma-Aldrich (St Luis, MO).

### Cell Culture

Human pancreatic cancer stem cells (CSCs) were cultured in either Celprogen's pancreatic CSC medium or DMEM supplemented with 1% N2 Supplement (Invitrogen), 2% B27 Supplement (Invitrogen), 20 ng/ml human platelet growth factor (Sigma-Aldrich), 100 ng/ml epidermal growth factor (Invitrogen) and 1% antibiotic-antimycotic (Invitrogen) at 37°C in a humidified atmosphere of 95% air and 5% CO_2_.

### Tumor Spheroid Assay

Spheroid forming assays were performed as described elsewhere [31], [80]. In brief, single cells were plated in six-well ultralow attachment plates (Corning Inc., Corning, NY) at a density of 1,000 cells/ml in DMEM supplemented with 1% N2 Supplement (Invitrogen), 2% B27 Supplement (Invitrogen), 20 ng/ml human platelet growth factor (Sigma-Aldrich), 100 ng/ml epidermal growth factor (Invitrogen) and 1% antibiotic-antimycotic (Invitrogen) at 37°C in a humidified atmosphere of 95% air and 5% CO_2_. Spheroid were collected after 7 days and dissociated with Accutase (Innovative Cell Technologies, Inc.). The cells obtained from dissociation were sieved through a 40-µm filter, and counted by coulter counter using trypan blue dye.

### Soft agar colony assay

Cell suspensions (2,500 cells) were prepared using 0.4% Noble agarose (Becton Dickinson) and overlayed onto a 60-mm dish containing a solidified bottom layer of 0.6% agarose in medium. Once the top layer solidified, 1 ml of medium was placed on top of the cell layer. After treatment, plates were incubated for 3 weeks and colonies were counted.

### Transwell Migration assay

For transwell migration assays, 1×10^5^ prostate CSCs were plated in the top chamber onto the noncoated membrane (24-well insert; pore size, 8 µm; Corning Costar) and allowed to migrate toward serum-containing medium in the lower chamber. Cells were fixed after 24 hours of incubation with methanol and stained with 0.1% crystal violet (2 mg/mL, Sigma-Aldrich). The number of cells invading through the membrane was counted under a light microscope (40×, three random fields per well).

### Transwell invasion assay

For invasion assay, 1×10^5^ cells were plated in the top chamber onto the Matrigel coated Membrane (24-well insert; pore size, 8 µm; Corning Costar). Each well was coated freshly with Matrigel (60 µg; BD Bioscience) before the invasion assay. Cells were plated in medium without serum or growth factors, and medium supplemented with serum was used as a chemo-attractant in the lower chamber. The cells were incubated for 48 hours and cells that did invade through the pores were removed by a cotton swab. Cells on the lower surface of the membrane were fixed with methanol and stained with crystal violet. The number of cells invading through the membrane was counted under a light microscope (40×, three random fields per well).

### Viral production and infection

Nanog shRNA construct (pLKO.1-puro, Mission RNAi) was from Open Biosystems. Lentiviral-TOP-dGFP-reporter (pRRL.sin-18.ppt) has been described elsewhere [81]. Lentivirus was produced by triple transfection of HEK 293T cells. Viral supernatants were collected and concentrated by ultracentrifugation to produce virus stocks with titers of 1×10^8^ to 1×10^9^ infectious units per milliliter. Viral supernatant was collected for three days by ultracentrifugation and concentrated 100-fold [82]. Titers were determined on HEK293T cells. Human prostate CSCs were transduced with a mixture of viral particles and polybrene with two rounds of infections [82].

### Caspase-3/7 Assay

Cells (3×10^4^ per well) were seeded in a 96-well plate with 200 µl culture medium. Approximately 16 h later, cells were treated with various doses of resveratrol. Casapse-3/7 activity was measured by a fluorometer as per manufacturer's instructions (Invitrogen).

### Western Blot Analysis

Western blots were performed as we described earlier [23], [83]. In brief, cells were lysed in RIPA buffer containing 1 X protease inhibitor cocktail, and protein concentrations were determined using the Bradford assay (Bio-Rad, Philadelphia, PA). Proteins were separated by 12.5% SDS/PAGE and transferred to membranes (Millipore, Bedford, MA) at 55 V for 4 h at 4°C. After blocking in 5% nonfat dry milk in TBS, the membranes were incubated with primary antibodies at 1∶1,000 dilution in TBS overnight at 4°C, washed three times with TBS-Tween 20, and then incubated with secondary antibodies conjugated with horseradish peroxidase at 1∶5,000 dilution in TBS for 1 hour at room temperature. Membranes were washed again in TBS-Tween 20 for three times at room temperature. Protein bands were visualized on X-ray film using an enhanced chemiluminescence detection system.

### Quantitative real-time reverse transcription-PCR

For the determination of gene expression levels, RNA was extracted from cells using trizol and treated with DNase I (Sigma). Total RNA (1 µg) was retrotranscribed using the ImProm-II Reverse Transcription System (Promega). SYBR Green I quantitative real-time reverse transcription-PCR (qRT-PCR) was performed, and the housekeeping gene GAPDH was used to normalize the variation in cDNA levels. SYBR Green I amplifications were performed using the GeneAmp 7000 Sequence Detection System (Applied Biosystems). Custom primers for Nanog, Sox-2, cMyc, ABCG2, Zeb1, Snail and Slug were purchased from realtime primers.com.

### Generation of KRAS^G12D^ Mice

LSL K-ras^G12D^ and Pdx-1-Cre mice were obtained from the National Cancer Institute (Frederick, MD). LSL K-ras^G12D^ mice were crossed with the Pdx-1-Cre mice to obtain *Kras^G12D^ (Pdx1-Cre;LSL-Kras^G12D^) mice.* The recombined Kras*^G12D^* allele was identified by PCR. *Pdx1-Cre;LSL-Kras^G12D^* mice develop early stage PanIN lesions at 2 months of age and the vast majority of ducts are normal [36]. However, mice develop statistically significant numbers of advanced PanIN lesions (stages 2 and 3) about 7 months, and the vast majority of ducts are abnormal [36]. Beginning at approximately this age, these mice also typically begin to develop invasive and metastatic pancreatic ductal adenocarcinoma.

### Statistical Analysis

The mean and SD were calculated for each experimental group. Differences between groups were analyzed by one or two way ANOVA, followed by Bonferoni's multiple comparison tests using PRISM statistical analysis software (GrafPad Software, Inc., San Diego, CA). Significant differences among groups were calculated at P<0.05.
